# The role of Negr1 in cortical development via NCAM-FGFR2 signaling

**DOI:** 10.1186/2193-1801-4-S1-P38

**Published:** 2015-06-12

**Authors:** Francesca Pischedda, Joanna Szczurkowska, Maria Daniela Cirnaru, Laura Cancedda, Giovanni Piccoli

**Affiliations:** University of Milan, Italy; Italian Institute of Technologies Genova, Italy; Università Vita-Salute, Italy

**Keywords:** Autism, Negr1, MAPK

Autism spectrum disorder (ASD) affects 0.9% of children and it is recognized as the most genetic of all developmental neuropsychiatric syndromes. Mutations in NEGR1 and FGFR2 genes have been recently identified as ASD candidates. Negr1 is a member of IgLON adhesion protein family but its functions are largely unknown. Our original approach has identified Negr1 as a developmentally regulated synaptic protein. Thus we examined the consequences of Negr1 acute downregulation. Strikingly, we found that Negr1 ablation impairs neuronal maturation in vitro1. A combination of biochemical and imaging investigation has demonstrated that Negr1 influences neurites outgrowth via MAPK signaling organizing trans-synaptic heterodimer. In detail, we demonstrated that ectopic Negr1 is sufficient to improve neurite arborization and rescue the morphological phenotype observed in Negr1 KD cells. This function is dependent on the activation of MAPK pathway through tyrosine kinase receptors. In fact, we found that Negr1 physically and functionally interacts with NCAM and FGFR2, modulates FGFR2 response to FGF and consequently influences MAPK pathway. FGFR2/NCAM pathway plays an important role during brain development. Not surprisingly, our investigation of the radial migration of newly generated cortical neurons, revealed that Negr1-FGFR2-NCAM cross-talk controls cortical organization in vivo. Connectivity dysfunctions have been suggested as causative alterations in ASD. Given the functional, physical and genetic correlation among Negr1 and FGFR2 and NCAM, the study of Negr1/FGFR2/NCAM molecular cross talk may offer new therapeutic opportunities.
